# Development of a Patient-Centred, Psychosocial Support Intervention for Multi-Drug-Resistant Tuberculosis (MDR-TB) Care in Nepal

**DOI:** 10.1371/journal.pone.0167559

**Published:** 2017-01-18

**Authors:** Sudeepa Khanal, Helen Elsey, Rebecca King, Sushil C. Baral, Bharat Raj Bhatta, James N. Newell

**Affiliations:** 1 Health Research and Social Development Forum (HERD) Nepal, Thapathali, Kathmandu, Nepal; 2 Nuffield Centre for International Health and Development (NCIHD), University of Leeds, Charles Thackrah Building, Leeds, United Kingdom; Foundation for Medical Research, INDIA

## Abstract

Multi-drug-resistant tuberculosis (MDR-TB) poses a major threat to public health worldwide, particularly in low-income countries. The current long (20 month) and arduous treatment regime uses powerful drugs with side-effects that include mental ill-health. It has a high loss-to-follow-up (25%) and higher case fatality and lower cure-rates than those with drug sensitive tuberculosis (TB). While some national TB programmes provide small financial allowances to patients, other aspects of psychosocial ill-health, including iatrogenic ones, are not routinely assessed or addressed. We aimed to develop an intervention to improve psycho-social well-being for MDR-TB patients in Nepal. To do this we conducted qualitative work with MDR-TB patients, health professionals and the National TB programme (NTP) in Nepal. We conducted semi-structured interviews (SSIs) with 15 patients (10 men and 5 women, aged 21 to 68), four family members and three frontline health workers. In addition, three focus groups were held with MDR-TB patients and three with their family members. We conducted a series of meetings and workshops with key stakeholders to design the intervention, working closely with the NTP to enable government ownership. Our findings highlight the negative impacts of MDR-TB treatment on mental health, with greater impacts felt among those with limited social and financial support, predominantly married women. Michie et al’s (2011) framework for behaviour change proved helpful in identifying corresponding practice- and policy-level changes. The findings from this study emphasise the need for tailored psycho-social support. Recent work on simple psychological support packages for the general population can usefully be adapted for use with people with MDR-TB.

## Introduction

Tuberculosis (TB) continues to be a significant threat to global public health, particularly in low- and middle-income countries where more than 95% of TB deaths occur [1]. Antibiotic resistance severely undermines global efforts to control TB. Globally, 5% of TB cases, 480,000 people, were estimated to have multi-drug-resistant TB (MDR-TB) in 2014 i.e. 3.3% of all new TB cases and 20% of previously treated TB cases. Among MDR-TB patients who started treatment in 2012, only 50% successfully completed treatment. Of the rest, 16% died, 16% were lost to follow-up, treatment failed for 10% and 8% had no outcome information [1].

The WHO-endorsed ‘Directly Observed Treatment Strategy-Plus’ (DOTS-Plus) strategy for MDR-TB includes treatment that commonly lasts 20 months but sometimes as long as 24 months [2]. While it has been shown that drug-sensitive TB treatment can successfully be delivered by community and family members [3], the nature of MDR-TB treatment raises challenges for effective delivery using this approach. Treatment for MDR-TB is long and unpleasant. An initial intensive phase consisting of daily injections lasts for eight months, although the duration may be modified according to the patient’s response to treatment [2]. One clinical challenge to MDR-TB treatment is the severity of drug side-effects: in particular, ototoxicity, sometimes leading to permanent hearing loss; gastrointestinal disturbance; and frequently psychiatric disorders [4,5]. These can not only lead clinicians to stop treatment, but also increase risks of patient non-compliance.

Whether as a consequence of drug side-effects or the challenges of living with MDR-TB and its treatment, depression and anxiety are common [4,6,7]. Given the extent of mental health problems faced by patients, there are surprisingly few studies that explore this important issue. This is particularly concerning given the apparent correlations between mental health problems and treatment completion and outcomes. MDR-TB patients with neurological side-effects have been found to have significantly worse outcomes and higher likelihood of death (odds ratio, 13.8; 95% CI 2.2–86.7) [5]. Furthermore, among drug-sensitive TB patients, poor quality of life persists even after cure [8], and the situation is likely to be much worse for MDR-TB survivors.

The social and economic impacts of MDR-TB on families, carers and the household have been explored in several qualitative studies. For household members, the burden of providing care and facing the stigma of TB leads to social isolation and poverty [9]. Household members caring for TB patients have poorer mental health and depression, with lower quality of life scores [10]. Given these psychosocial and economic challenges to patients and their households, it is unsurprising that non-completion rates remain high for MDR-TB. A recent systematic review found MDR-TB treatment non-completion rates ranged from 0.5% to 56%, with a pooled rate of 14.8% (95%CI 12.4–17.4) [11].

A key pillar of WHO’s Stop TB Strategy [12] and post-2015 End TB strategy [13] is integrated patient-centred care. WHO’s Standard 9 for TB Care [14] identifies the purpose of this as “to promote adherence, improve quality of life, and relieve suffering”. Research provides indications of specific patient-centred interventions that may be effective. The Toczek et al (2013) review identified several strategies associated with lower non-completion rates, including the use of community health workers to support patients throughout treatment, managing smaller cohorts of patients, providing patient education and a comprehensive package of adherence interventions (including financial support, transport reimbursement, food and nutrition). No significant differences were found between programmes that did and did not offer counselling [11]. Given the commonality of mental health issues among MDR-TB patients, it is surprising that that no significant differences were found. However, ‘counselling’ is used to describe a wide range of interventions, and it may well be that those included in Toczek’s review were limited in their design and implementation. We found little published research on the effectiveness of carefully developed and sensitive psychosocial support. However, pilot work in Nepal indicates that the provision of counselling alone and in combination with financial support may improve cure rates [15]. Likewise, a study in Peru, describing a programme of patient support groups facilitated by psychologists and health workers, found that of 285 patients who received the intervention only 3.5% defaulted [16]. The role of nurses in providing emotional support to MDR-TB patients and their ability to encourage treatment adherence has also been noted [17]. We found no studies that assess the impact of interventions for MDR-TB patients on mental health or on quality of life.

The value of maintaining quality of life throughout treatment for MDR-TB goes beyond minimising default rates or even maximising cure rates, as health professionals also have a responsibility to reduce suffering. In light of this we decided to develop and test a psychosocial support package that can be used within routine care for MDR-TB patients in Nepal. This paper reports the first two phases of this work. The first phase used qualitative methods to investigate, from the perspectives of patients, family members and health workers, the challenges patients face throughout their treatment for MDR-TB and how these challenges determine patients’ psychosocial wellbeing. The second phase focused on developing an intervention that could be delivered with the NTPs existing programme. Intervention development was informed by the determinants identified in phase one, in conjunction with current evidence and expert opinion. Expert opinion in this case refers to the opinions of patient, family members, NTP staff and those working with MDR TB patients. Michie et al’s (2011) Behaviour Change Wheel has been used to provide a framework to link the determinants of psychosocial wellbeing to components of the intervention. The implementation of this package is currently being tested in Nepal.

## Methods

This study received ethical approval from:

1. Nepal Health Research Council, RamShah Path, Kathmandu, PO Box 7626, Nepal
2. Faculty of Medicine and Health Research Ethics Committee, University of Leeds, Worsley Building, Leeds, LS2 9LJ

### Study setting

In Nepal, WHO estimates that in 2013 the MDR-TB burden was 1110 cases; NTP records report 477 laboratory-confirmed MDR-TB cases, of which 388 started on treatment [18]. Treatment for MDR-TB is delivered through 13 treatment centres, where initial diagnosis and clinical management takes place, and 71 sub-centres, where patients must go daily to receive their medication. MDR-TB clinic in-charges are given a 5-day modular training course on MDR-TB, which includes information to be given to patients about the disease, its treatment, the side-effects of drugs and what patients should do if they experience any side-effects. Nepal is divided into three ecological zones: mountain, hill and plains. For those in mountain and hill zones, access to treatment centres or even sub-centres can involve several days journey. For many, relocation near to a treatment centre during the 20 months of treatment is the only viable option. In light of these constraints, the NTP currently provides 8 hostels for MDR-TB patients close to treatment centres. These hostels have capacity for 12 to 15 patients. In addition, the government provides 1500 Nepalese Rupees (approximately US$10) every month to every MDR-TB patient.

### Qualitative study

#### Study design

We used qualitative methods to investigate the challenges faced by adult (over 18 years) patients during their treatment for MDR-TB. We conducted semi-structured interviews (SSIs) and focus group discussions (FGDs) with MDR-TB patients, their family members and health workers. We purposively selected participants from two treatment centres in the Central and Western regions and one sub-centre in the Central region. We conducted interviews between August and December 2012. We chose this mix of centres and sub-centres to ensure a variety of patients from different geographical locations, ethnic and wealth backgrounds and to increase the transferability of our findings to other areas in Nepal. We purposively sampled MDR-TB patients treated at these sites to provide variety in terms of age, gender and caste. In addition, we purposively selected one hostel where we conducted SSIs with resident patients.

We took a systematic, iterative approach to the development of the SSI guides, which consisted of around 13 open questions, with prompts to follow up on particular issues of interest. We carried out three pilot interviews, transcribed recordings, reflected on the process, and revised the interview guides accordingly. We followed a similar process for the FGD guides. The interview guides explored the physical, psychological and social experience of MDR-TB and its treatment from the perspectives of patients, family members and health workers. They also explored service delivery factors that were perceived as impacting on the patient and family member well-being.

Data collection, analysis and intervention development was coordinated by HERD, a Nepalese health research and development NGO that has worked in the TB sector for many years.

#### Data collection

Interviews were conducted in the premises of the DOTS-Plus Centres in a place where there was adequate ventilation (to minimise transmission) and privacy. Interviews with patients and health workers were conducted by a female Nepalese researcher (SK) with post-graduate training in qualitative research methods and experience in conducting SSIs in health service contexts in Nepal. We audio-recorded interviews in Nepali and took detailed hand-written notes. Interviews lasted for approximately one hour. FGDs with patients and family members were held in training halls or meeting rooms at the centres/sub-centres. After initial introductions, the staff of the centre kept away from the interviews and FGDs to allow participants to speak more freely about every aspect of their care. The FGDs were facilitated by SK, two other qualitative researchers and a programme manager with extensive TB experience from HERD. We audio-recorded FGDs in Nepali and a dedicated note-taker took detailed hand-written notes. Throughout the discussion we wrote the key points raised by the groups on flip charts in Nepali and ensured that participants were satisfied with our understanding. SK subsequently transcribed the interviews and FGDs and translated them into English. Information sheets and consent forms were provided to all participants. Written consent was obtained from those who could provide it. If participants could not read and write, the information sheets and consent forms were read out to them and a thumb-print was taken.

#### Data analysis

We analysed the interview and FGD transcripts and the FGD notes and flip charts using the Framework Approach [19]. We chose this approach as it allows deductive analysis based on pre-identified objectives, as well as the emergence of themes inductively from the data. Three members of the research team (RK, HE, SK) independently coded a sample of transcripts and, through detailed discussion, agreed a coding frame, including both a-priori and emergent codes. SK coded the remaining transcripts and discussed any amendments to the coding frame with RK and HE. We organised the data into charts, which allowed analysis of responses by type of respondent. We carried out further detailed analysis (a) to explore the effect of gender and relocation status (which had emerged as important respondent characteristics) on responses and (b) to investigate theories to explain the responses given. We then assessed our findings using the Behaviour Change Wheel [20] to identify possible capabilities, opportunities and motivators (COM-B) for health workers and patients to change their behaviour to enable patients to have an acceptable quality of life while ensuring they complete treatment.

#### Limitations

We were only able to recruit patients who were currently on treatment; clearly, patients who were unable to complete MDR-TB treatment would have offered invaluable insights into the psychosocial challenges of remaining on treatment. All interviews and FGDs were conducted within the premises of the health centre, so participants may have felt hesitant to express dissatisfaction with the service. However, health workers complied with our requests not to stay within earshot of the interviews and FGDs.

### Intervention development

Following the completion of phase one, we structured the intervention development phase to enable government ownership, to increase the likelihood of national scale-up and sustainability of the intervention [21]. The intervention development phase started in October 2013 and lasted until December 2014. We engaged health workers, MDR-TB patients and their family members in the development of the intervention to ensure the feasibility and appropriateness of the proposed intervention. We worked with these stakeholders over a nine-month period, holding ten meetings within the HERD team; eight with the NTP; four with NGOs delivering TB services; seven with an NGO providing psychosocial support; seven with the National Health Information Education Communication Centre (NHEICC); eight with patients; and we held two technical working group meetings with TB experts. Finally, key to shaping the intervention, we conducted a national workshop to (a) check the findings and interpretation of the study made sense to providers, patients and their family members and (b) ensure that the proposed package could be easily implemented and sustainable. The workshop was held in April 2014 in Kathmandu and included representatives from the NTP (including the NTP Director), the NHEICC, MDR-TB patients and their family members, WHO representatives, NGO TB service providers and HERD staff.

## Results and Discussion

### Characteristics of the respondents

SSIs were held with 15 patients—10 men and 5 women, aged 21 to 68 (Table 1). Four family members of the interviewed patients were sampled for individual interviews. Three of these were mothers of patients and one the wife of a patient (Table 2). Three frontline DOTS providers were interviewed across the three different districts, two male and one female. All the providers were responsible for DOTS-Plus within the particular health facility. Two had over 6 years of experience working in the DOTS-Plus centre and one had two years of experience. In addition, two FGDs were held with MDR-TB patients, two with their family members and one was a mixture of the two (Table 3). The first FGD combined the two groups. However, we found that participants were reluctant to discuss the issues they faced in the presence of their relatives. Therefore, we conducted the remaining FGDs separately.

**Table 1:** Characteristics of patients who participated in individual interviews in three regions in Nepal (September 2012 to September 2013).

Patient ID number	Age	Gender	Education level	Occupation	Region	Type of case	Family member interviewed	Living arrangements
P1	32	M	Higher	Shopkeeper	Central	Relapsed	No	Home with family
P2	28	M	Secondary	Student	Eastern	Relapsed	No	Hostel
P3	35	M	Secondary	Businessman	Central	Relapsed	No	Rented room alone
P4	32	F	Illiterate	Laborer (daily wage worker)	Central	Relapsed	No	Rented room alone
P5	21	F	Primary	Student–now dropped out	Central	Relapsed	Yes (mother)	Rented house with mother
P6	32	F	Primary	Laborer (daily wage worker)	Central	Primary infection with MDR	No	Rented room with children
P7	26	M	Primary	Unemployed	Western	Relapsed	Yes (wife)	Rented room with wife
P8	22	F	Higher	Student	Midwest	Primary infection with MDR	Yes (mother)	Home with family
P9	32	M	Primary	Laborer (daily wage worker)	Western	Relapsed	No	NGO hostel
P10	58	M	Primary	Retired Indian army	Western	Relapsed	No	Home with family
P11	68	M	Primary	Retired Indian army	Western	Relapsed	No	Rented house with family
P12	33	M	Primary	Security guard	Western	Relapsed	No	Rented room alone
P13	25	M	Higher	Student–now dropped out	Western	Relapsed	No	Home with family
P14	18	M	Secondary	Student–now dropped out	Western	Relapsed	No	Home with family
P15	23	F	Higher	Student–now dropped out	Western	Primary infection with MDR	No	Home with family

**Table 2:** Characteristics of family members who participated in individual interviews in three regions in Nepal (September 2012 to September 2013).

Family member ID number	Corresponding patient	Relationship with the patient	Age (estimate)	Education status	Occupation
FM1	P5	Mother	35	Illiterate	Sweeper
FM2	P7	Wife	18	Secondary	Housewife
FM3	17 year old female patient (too young to consent to be interviewed)	Mother	35	Primary	Housewife
FM4	P8	Mother	50	Primary	Family business

**Table 3:** Patient and family member participants in focus group discussions in three regions in Nepal (September 2012 to September 2013).

Focus Group	Patient group or family member group	Number of males/females	Where FGD held
1	Patient group	Male: 4 Female: 1	Sub-treatment centre, Kathmandu
2	Family members group	Male: 1 Female: 5	Sub-treatment centre, Kathmandu
3	Patient group	Males: 4 Female: 2	Zonal hospital (treatment centre)
4	Family members group	Males: 4 Female: 1	Zonal hospital (treatment centre)
5	Combined group of patients and family members Patient Family members	Males:7 Females:2 Males: 5 Females: 4	Sub-treatment centre, Kapilavastu

### Key themes emerging from the analysis

#### Theme 1: physical and psychological impact

The physical health of all the patients interviewed was greatly impacted by MDR-TB and its treatment. All patients identified physical side-effects from their MDR-TB treatment, in particular knee and other joint pain, hearing problems, eye problems, and loss of appetite. All family members who were interviewed commented particularly on the loss of appetite and reduced mobility. These effects were confirmed by health workers.

The psychological impacts of MDR-TB and its treatment dominated many of the interviews with patients. It was difficult to delineate whether these were directly due to the medication or to interactions with wider socio-economic conditions. When associated with the medication, these feelings ranged from confusion and limited mental ability to ‘going mad’:

*“I think these medicines are making me go mad*. *Others may not experience the same thing*, *but it was bad for me*. *It made me feel very unstable–I would feel one thing one minute*, *and something else the next*, *and be unable to make any decisions*.*”* (P2)

The physical and psychological side-effects of the drugs and this lack of knowledge fuelled patients’ anxiety and led to a feeling of hopelessness, particularly those in the early phases of treatment:

*“I think there is a 90% chance that I will die*. *There is only a 10% chance that I will survive*. *I cannot even go to the toilet*. *I need support*. *When I eat*, *I immediately throw up*. *When I take my medicines*, *I immediately throw up*.*”* (P2)

The level of confidence patients had in the effectiveness of the drugs also influenced their sense of hope and belief in being cured. Patients who had seen others dying from MDR-TB found it particularly difficult to maintain hope:

*“I think about the future*. *I have MDR-TB now and I think about my children*. *I have only seen people dying from MDR-TB*. *I have not seen anyone surviving*.*”* (P3)

#### Theme 2: health service facilities, information and the psychological impact of MDR-TB

Patients and family members generally lacked knowledge about MDR-TB, its treatment and side-effects. The majority of respondents did not know about MDR-TB before their diagnosis. Some had heard about ‘normal’ (i.e. drug-sensitive) TB from their family or acquaintances that had had TB, while others thought TB was caused by poor diet or hard work. However, one family member was more knowledgeable, and this seemed to be connected to his experience of another relative having MDR-TB.

Limited knowledge about MDR-TB and its treatment seemed to fuel patients’ anxieties. Those who had previously been treated unsuccessfully for drug-sensitive TB were particularly fearful and lacked any faith in the potential for MDR-TB treatment to cure their disease. Many patients and their carers expressed their anxieties in knowing what side-effects to expect and how to deal with them if they occurred. This lack of knowledge also meant that patients were not clear whether they should seek medical help, whether they were indeed experiencing a side-effect or a symptom of MDR-TB and whether the side-effect would pass. Inadequate information and the concerns and confusion it engendered led to further psychosocial stress for patients and their family members.

Some health workers said they gave information to patients, but interviews with patients showed their inadequate understanding of the causes and management of MDR-TB. Both health workers and patients perceived that health workers had little time available to counsel patients. This perception inhibited patients from asking health workers for information and advice. Patients who did get advice were generally more educated, and such people also appeared to have access to other sources, with the younger patients identifying books and internet searches as ways to find advice or information about TB. When information had been given on transmission and effective infection control procedures, the impact on patients was clear:

*“I used to think that I will never get married*, *because my children will also get MDR-TB*. *However*, *I read on a poster that this is not the case*. *It would help if we had access to more information*.*”* (P2)

There was also evidence that in one treatment centre, health workers were exploring ways to provide timely support and advice to patients by encouraging patients to phone the centre to discuss any problems.

The physical infrastructure of some DOTS-Plus centres led to some patients to complain of lack of privacy. In one centre in particular the lack of consultation rooms was a severe constraint leading to treatments, including injections into the buttock, being given in view of other patients

#### Theme 3: patients’ contact with family and wider social networks

MDR-TB and its treatment impacted on the patients’ contacts with family and wider social networks and this, in turn, impacted on their psychosocial well-being.

Fear of infecting others frequently undermined patients’ continued engagement with their families and wider social networks. Notably, most patients had good knowledge of infection risk and infection control. Some patients reported distancing themselves from their families due to the fear of transmitting the disease to others. Younger patients seemed particularly affected by this restriction on their social networks:

*“I have to stay away from my friends*. *I feel uncomfortable because I might transmit it to other people*. *I used to spend time with my friends*, *but I can’t do that because I am aware that I have MDR-TB and I could infect my friends*. *This makes me feel very scared*.*”* (P13)

Several participants also explained how relatives stopped visiting them because of fear of transmission and this added to their emotional stress. Family members also commented on how the fear of infection affected family relations for patients:

*“His own family refuses to sit next to him in case they catch TB*. *When people find out that he has TB*, *they discriminate against him…His elder brother has a daughter and she is not allowed to come anywhere near my husband*. *If she does*, *her mother tells her off*.*”* (FM 2)

However, not all participants reported stigma within the family:

*“No*, *there has not been any change…She is my daughter*, *so why would I behave differently with her…Neither my daughter nor I have noticed any changes in the behaviour of our family members*. *Her brothers and aunts come and visit and treat her in the same way*. *They are understanding*. *There has not been any tension in the family*.*”* (FM 3)

Despite the difficulties faced, families often provided crucial support to MDR-TB patients. Most of the patients acknowledged the role of family members in the treatment process. Parents and wives, in particular, were heavily involved in day-to-day care including providing food, accompanying patients to the treatment centre, facilitating discussions with health workers and most importantly from the patients’ points of view, providing psychological support. For those living with their family members, the value of the support they received was overwhelmingly evident and the support of wives and mothers was a common theme for male participants. In particular, married men often had very strong support from their wives. Some of the family members who were taking care of the patients were aware of the vital role they played in supporting the patient, particularly in the early months of treatment:

*“My parents encouraged us to separate*, *but I was determined not to leave him*. *I don’t think it is right to be with someone when he is well and then leave him when he is unwell*. *I chose to be with him and to put some distance between us and my parents…I spend my whole day cooking for him*, *making sure he takes his medicines and washing his clothes*.*”* (FM 2)

Family involvement in the treatment process was considered essential, and parents of younger MDR-TB patients were particularly involved in their treatment:

*“There was no question of her going on her own*. *She couldn’t even stand up*. *We had to bring her for her treatment*. *Now*, *her health is improving*. *Sometimes I accompany her for her treatment and sometimes her father does…I would be very worried to send her on her own”*. (FM3)

However, some patients complained that their family members did not provide enough support while they underwent treatment. This was mainly the case of married women who were either the subject of discrimination or were forced to leave their homes by their husbands after being diagnosed with MDR-TB.

Likewise, health workers confirmed the challenges faced by MDR-TB patients in relation to family networks, but indicated that the impact varied depending on the relationship between the patient and the family members. In most cases, parents were found to be supportive of their children; wives and mothers-in-law were frequently identified as lacking in support.

*“We often see family members abandoning people who have TB*, *but those who are close to them continue to support them…Usually*, *parents do support their children*, *but that is not always the case when it is a different family member*.*”* (HW1)

#### Theme 4: living arrangements

Patients’ living arrangements were determined by access to treatment centres, concerns about transmitting MDR-TB, marital status and gender. Those living far from treatment centres were forced to relocate. Married male patients predominantly lived with their wives and families even if this meant relocating the whole family. Some men stayed in a hostel for the duration of their treatment, but had regular contact with their families. Hostels were occupied mainly by male patients who could be either single or married, but were living away from their families. This made it difficult to accommodate female patients, so very few women were found in hostels. Married women generally lived alone in rented accommodation. Younger single patients, whether male or female, were predominantly living with their parents and wider extended families.

Many respondents had faced challenges in finding rooms to rent and being evicted once their MDR-TB status was discovered. Particular challenges were faced by married women who had been abandoned by their husbands and families:

*“Some people say that they do not want to rent a room to a single woman*, *others say that the room has already been rented out*, *and others say that they will not rent it to people who are sick*.*”* (P4)

For these women, living alone with no support was even more challenging when they also had children to support:

*“When I lived with my family*, *they prepared food for my children*. *Now that I am alone*, *I have to take care of their food and all their other needs*, *regardless of how I am feeling*.*”* (P3)

These different living arrangements and the extent to which patients were supported by their families played a key role in their wellbeing. Those who lived away from their families (whether having relocated for treatment or because of discrimination) were clearly affected by a lack of social support. However, patients who had to relocate for treatment purposes often mentioned support from their families in the form of cash. Patients living in hostels also reported a lack of social support and reduced well-being. It was evident that health workers were not able to fill this gap:

*“We don’t meet the doctors and the sisters don’t have time to talk to us*. *So who should we talk to*? *…*. *I do nothing*. *I sit in the corner and cry a lot*. *There is no-one to talk to*.*”* (P4)

#### Theme 5: financial circumstances and livelihoods

Almost all the patients said MDR-TB had a negative impact on their livelihood. Many patients had stopped work or education due to their poor physical health and the need for daily visits to the treatment centre. The influence on mental health and well-being was evident:

*“I don’t work because I can’t work*. *I can’t lift heavy things because I experience pain in my joints*. *I don’t have the energy to work anymore*. *I used to work in [a manual job]*…*I have not looked for another job*. *My biggest problem is my inability to work*.*”* (P3)

For the poorest, the inability to work undermined not just their mental health but also their basic survival. This was particularly the case for married women who had been abandoned by their husbands and families. Where these women were also looking after children, the effects of not being able to work were catastrophic:

*“I can't work either*. *I run a small business selling beaten rice*, *but it doesn’t do well*. *I only earn Rs100-150 [about $1] a day*. *How will that money help*? *Should I pay rent with that money or eat*? *It is not sufficient*.*”* (P 4)

Patients also emphasised that the need to relocate for treatment caused major hardships with increased costs due to room rental and higher living expenses.

Health workers also confirmed the financial burdens faced by patients:

*“Most of the patients complain about the financial difficulties they face due to the treatment*. *Even the local patients complain that*, *as they have to attend the health centre every day*, *their business is adversely affected*.*”* (HW 2)

While most patients, irrespective of their area, location, treatment duration and socioeconomic condition, knew the NTP provided Rs1500 each month, many identified problems with the frequency, regularity and mechanism to receive this money:

*“We have to go to Thimi [treatment centre] to receive the monthly allowance…But so far they have not given it to me and…I do not know…It’s not regular*.*”* (P8)

Family members also commented on difficulties with the amount and the disbursement of money.

As described above, the need to relocate, whether to rented accommodation or a hostel, to be nearer to a treatment centre, greatly influenced the well-being of patients. However, for all patients the need to attend the treatment centre on a daily basis to receive their treatment presented challenges. Most patient respondents were daily wage workers, so the burden of attending the clinic meant the loss of that day’s income. For many the daily transport costs had to be paid from the 1500 NRS ($10) paid each month to MDR-TB patients by the NTP. Many were aware that this money was supposed to provide them with a more nutritious diet; however the financial strain meant that the money was rarely used for this purpose. Consequently, patients became financially dependent on their families and those without family support struggled to survive.

### A framework of determinants of psychosocial wellbeing among MDR-TB patients

**Fig 1:**
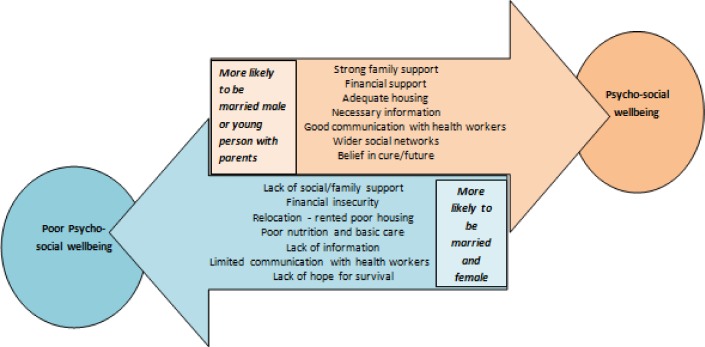
Framework for understanding the determinants of psychosocial wellbeing in MDR-TB patients in Nepal.

These findings shed new light on the influence of gender and marital status on patients’ abilities to maintain acceptable levels of physical and mental wellbeing throughout the challenging period of MDR-TB treatment. Our findings also concur with previous literature identifying the psychological, economic and social stress placed on family members [9,10,15,22,23] and the mental health impacts of MDR-TB [4,5], particularly in relation to drug side-effects [6,7]. Fig 1 illustrates how marital status and gender identified by patients and their families combine to either enhance or undermine the psychosocial wellbeing of those undergoing MDR-TB treatment.

#### Developing the intervention

The intervention development phase, led by HERD, drew on the qualitative findings, a review of the evidence base, and expert opinion to identify intervention components to address the determinants of psychosocial wellbeing.

While the advice of experts could be gained through the series of meetings described in the methods section above, the HERD team were aware that lay experts were unlikely to speak openly in front of health staff and managers. Patients and their family members did participate in the national workshop. However, to ensure their views on the content of the intervention were captured more thoroughly, they also provided their ideas at the end of the interviews and focus groups conducted during the qualitative study. The view of firstly, patients and carers, and secondly, healthcare and NGO providers and managers are detailed below:

*Patient and carer views*: To address anxieties about treatment and side-effects, both patients and carers highlighted several key recommendations for improving their care and experience. These were: clear information provided by health workers; clearly-defined referral routes in response to side effects; privacy during treatment, with participants emphasising that this could be achieved even within current facilities. To address issues of relocation and travel for treatment, participants suggested the provision of medicines, both injections and tablets, for several days or weeks for patients to take at home. For those who had relocated for their treatment many advocated that treatment should be provided within their home districts; failing that they advocated greater provision of hostel places. The majority of patients were keen for greater involvement of their family members. This included information targeted at families about MDR-TB and the course of treatment; it was hoped that this would help to address issues of stigma. Several participants felt that campaigns at community level were also required to reduce levels of stigma. All family members interviewed commented that the financial incentive was insufficient, particularly as a means to improve nutrition.

The analysis showed that patients providing suggestions were frequently the better-educated and male participants. The most vulnerable patients identified in our analysis made fewer suggestions. The most vulnerable patients were invariably those who had had to move away from home, particularly married women. Our qualitative analysis went some way to ensuring that the voices of these women were heard. Stressing these gender issues to participants at the national workshop meant that the experiences of these women were highlighted. This kept all other participants focused on the need to ensure the intervention was designed to support those most vulnerable to the psychosocial impacts of MDR-TB.

*Healthcare and NGO providers and managers*: The recommendations from health workers during the qualitative interviews, intervention design meetings and the national workshop were similar to those of patients and family members. In particular, they focused on campaigns to reduce stigma and decentralise services. Some health workers suggested vocational training interventions to improve patients’ financial circumstances during and after treatment. NGO providers shared the challenges of discussing treatment and side-effects to newly-diagnosed patients who are frequently in shock and unable to absorb information. Carers, predominantly family members, were seen as key in supporting the patient at this time. The qualitative findings triggered discussions on how best to target those most vulnerable, particularly married women. All agreed with the need to provide psychological counselling for patients. Spreading counselling sessions beyond the initial few days of diagnosis was seen as an important strategy, although it was debated whether a pre-specified number of sessions should be provided at fixed time-intervals or whether a more flexible approach throughout treatment was more appropriate. Central, district and health facility level NTP staff expressed concerns about adding psychosocial support to the existing heavy workload of staff. There was much discussion on the possibility of having additional counsellors allocated to DOTS-Plus centres to provide the psychosocial support needed.

Following the intervention development phase, the research team found Michie et al’s (2011) Behaviour Change Wheel a useful tool to structure the elements of the intervention proposed to ensure the determinants of psychosocial well-being were addressed. The Behaviour Change Wheel consists of a central “spoke” that enables the identification of determinants of a behaviour in terms of an individual’s capability, opportunity and motivation to perform it. The outer two “spokes” enable the identification of interventions and policies to address the determinants of the behaviour. This is a useful tool to use when identifying appropriate approaches to behaviour change. This process also highlighted how some intervention areas advocated by patients, carers and providers would require major policy and structural changes within NTP’s programmes, in particular the need for decentralised treatment services closer to patients’ homes. The close working relationship with NTP has enabled on-going input into policy development to ensure these aspects can also be considered.

*The intervention*: Through discussion with stakeholders, it was decided that selected areas of the proposed intervention would be tested initially due to resources available. Michie et al’s (2011) framework helped the team to carefully consider the purpose of each component of the intervention and this has helped to inform the content of training and materials. Table 4 below presents the components of the intervention as they relate to the relevant categories within Michie et al’s (2011) framework. The highlighted components will be tested within a feasibility study; those not highlighted are aspects to be considered at a policy level within NTP.

**Table 4:** Interventions considered to strengthen the psychosocial support for MDR-TB patients in Nepal, (2014).

Interventions (Michie et al 2011)	Intervention targeted at health workers and the health system	Intervention targeted at patients/family members	Purpose, determinant and COM-B addressed
Education: increasing knowledge and understanding.	**Developing understanding of different patient needs based on gender and living conditions through training.** Continual professional development and **guidelines for health workers on treatment, side-effects**.	**Leaflets, posters and session with health worker using flip book of key messages on side-effects, treatment process and infection control. Talks by health workers during peer support sessions.**	To increase knowledge on treatment and side-effects. Capability: psychological. Motivation: reflective.
Persuasion: using communication to induce positive or negative feelings or stimulate action.	**Role play during training of supportive relationship with patients. Posters presenting a positive image of compassionate health workers.**	**Inclusion of ‘expert patients’ successfully treated for MDR-TB to tell their stories during support groups and in leaflets. Use of buddying system to provide support to both patients and family members.**	To improve communication with health workers and build hope. Motivation: reflective and automatic.
Training: imparting skills.	**Training on communication skills and psychological counselling for health workers.**	**Health worker -led consultation with patients and carers using flip book to impart skills for dealing with treatment, side-effects, infection control, nutrition and basic care. Leaflets detailing the severity of side-effects, clarifying which require immediate clinical advice and which are less urgent and do not require treatment change. Access to a phone help-line to provide timely advice to patients and carers.**	To build skills of carers, patients and health workers. Capabilities: physical and psychological.
Environmental restructuring: changing the physical or social context.	Restructure clinics to allow privacy for patients to receive treatment and counselling. **Establishing phone help lines increasing HW opportunities to engage with patients within busy workload. Encouraging family members to attend appointments with patients throughout treatment.**	**Peer support groups for patients, and separately for family members to share problems and solutions. Ensuring that peer groups and buddying networks are geographically close and appropriate to the patient.**	To increase social support, particularly targeting those most in need. Motivation: automatic Opportunities: physical and social
Modelling: providing an inspirational example.	Awards for most supportive staff and DOTS Plus centres with case studies of their approach.	**Expert patients (men and women) providing example of living through MDR-TB. Posters showing supportive family, particularly with married women as patients.**	To build hope among patients and family member carers. Motivation: automatic.
Enablement: increasing means/reducing barriers.	Bringing care closer to patients, decentralised treatment centres. Use of disaggregated data to understand outcomes for different patient groups. Providing staff with the time/facilities/resources for patient support strategies and supportive monitoring and supervision. **Routine inclusion of assessments of social support, depression and anxiety during initial consultations and throughout treatment.**	Review of financial support amount and timely distribution. Review of hostel provision, with special consideration of women patients. Employment and livelihood opportunities to promote social inclusion, reduce stigma, and enhance access to services through economic support.	To provide a health system that enables targeted psycho-social support and reduces patient hardship. Capabilities: physical and psychological. Motivation: automatic Opportunities: physical and social.

*Text in bold indicates intervention strategies to be tried in the subsequent feasibility study. Non-highlighted text indicates areas of policy change for consideration by NTP.

This feasibility work will enable the team to identify which parts of the package work best, clarifying the who, what, where and when of delivery. Two key components of the intervention to be tested in the feasibility study within routine services include: 1) training and materials for health workers to provide clearer information on treatment and side-effects to patients and family members throughout treatment; and 2) assessment of all patients for depression and levels of social support at baseline and as part of the intervention evaluation. These two assessments are seen as key to tailoring the intervention to respond to the differing needs of patients. Based on the depression assessment results, depressed patients will be provided with psychological counselling delivered by health workers. The feasibility study will assess the practicality and success of offering patients a range of other support mechanisms including peer support groups (for patients and family members separately), a telephone help-line, a buddying system and ‘expert patient’ advice. The intervention will also test the training programme for health workers developed in this phase. In particular we will explore the extent to which the training methods (such as the use of vignettes and role plays) can help health workers to respond to the needs of married women.

## Conclusion

This study highlighted the limited provision of information on MDR-TB disease, treatment and drug side-effects to patients and their carers. This lack of knowledge contributed to patient anxiety. The extent of family and social support was a key determinant of psychological wellbeing. The extent to which this support is available was frequently determined by gender and marital status. Within this study, these determinants were used along with available research evidence and the tacit and programme knowledge of TB experts in Nepal to inform the development of an intervention to improve the psychosocial support of MDR-TB patients. The Behaviour Change Wheel framework proposed by Michie et al (2011) provided a useful framework for identifying intervention strategies. It also helped guide the content development of each intervention strategy to strengthen the capabilities, opportunities or motivations of patients, their family members and health workers to maintain psychological wellbeing throughout treatment. recognition of the need for a tailored approach taking into consideration the particular vulnerabilities of patients with limited social support who face high levels of stigma was key to informing the design and content of the intervention, which is currently being tested in Nepal.
